# Dr. R. H. L. Bibb

**Published:** 1890-07

**Authors:** 


### DR. R. H. L. BIBB.

In April last, the press dispatches announced the killing of Dr. Carothers, in Dr. Bibb's office, in Saltillo, Mexico, and stated that at the time the two gentlemen were alone. Commenting on it, the reporter intimated that there were grounds for suspecting Dr. Bibb of the killing. The same dispatch announced his arrest. A later telegram announced his release on bond. The JOURNAL was just going to press when the news came, and Dr. Bibb being a member of the Texas State Medical Association, and very popular throughout the State, the JOURNAL printed the item just as it was given to the daily papers, as an item of news of general interest to the profession. It transpires that Dr. Bibb and Dr. Carothers were intimate friends, and had agreed to go into a copartnership shortly, and that they were talking the matter over in Dr. Bibb's office. Not alone, however, as was alleged, but Dr. Bibb's servant, a negro man, was in the room at the time. Dr. C., like most young men, was fond of a joke, and he frightened the darkey with his pistol, in a playful way. The darkey became so seriously alarmed that to reassure him Dr. C. pointed the pistol at his own head, and by some strange fatality it was discharged, killing him; but not instantly, as stated. He lived long enough to exonerate Dr. Bibb, and the statement of the servant, the witness, corroborated it. Of course Dr. Bibb was arrested, as was every inmate of his household, even the cook; that is the law. But an examination was had as soon as possible, and Dr. Bibb was dismissed—not under bond, but fully exonerated of even a suspicion of crime.

The above statements we could substantiate were it necessary. We make them in justice to our friend and confrere, having, in publishing the dispatches, unintentionally done him a wrong and an injury. No one regretted the occurrence, or more seriously mourned the death of his young friend, than he. Dr. Bibb is a brave and chivalrous Southern gentleman, who would scorn a cowardly act; his honor is dearer to him than life. We tender him our sincere condolence in the bereavement of his genial young friend, and assure him of our continued high regard and esteem.

